# The Combination of Increased Temperatures and High Irradiation Causes Changes in Photosynthetic Efficiency

**DOI:** 10.3390/plants10102076

**Published:** 2021-09-30

**Authors:** Antonela Markulj Kulundžić, Marija Viljevac Vuletić, Maja Matoša Kočar, Anto Mijić, Ivana Varga, Aleksandra Sudarić, Vera Cesar, Hrvoje Lepeduš

**Affiliations:** 1 Agricultural Institute Osijek, Južno Predgrađe 17, 31000 Osijek, Croatia; maja.matosa@poljinos.hr (M.M.K.); anto.mijic@poljinos.hr (A.M.); aleksandra.sudaric@poljinos.hr (A.S.); 2 Faculty of Agrobiotechnical Sciences Osijek, J. J. Strossmayer University of Osijek, Vladimira Preloga 1, 31000 Osijek, Croatia; ivana.varga@fazos.hr; 3 Centre of Excellence for Biodiversity and Molecular Plant Breeding, Faculty of Agriculture, University of Zagreb, Svetošimunska cesta 25, 10000 Zagreb, Croatia; 4 Department of Biology, J. J. Strossmayer University of Osijek, Ul. cara Hadrijana 8/A, 31000 Osijek, Croatia; vera.cesar@biologija.unios.hr; 5 Faculty of Dental Medicine and Health, J. J. Strossmayer University of Osijek, Crkvena 21, 31000 Osijek, Croatia; hlepedus@ffos.hr; 6 Faculty of Humanities and Social Sciences, J. J. Strossmayer University of Osijek, Ul. Lorenza Jagera 9, 31000 Osijek, Croatia

**Keywords:** chlorophyll *a* fluorescence, enzyme, *Helianthus annuus*, irrigation, solar radiation, temperature

## Abstract

Global warming and the associated climate change are imposing abiotic stress on plants. Abiotic factors are crucial for plant productivity, survival, and reproduction. Eight sunflower hybrids were tested in conditions of different water availability and with combinations of different temperatures and irradiation. The changes in the photosynthetic efficiency were measured in the morning (control conditions: 2013, 25.8 °C and 349.1 W m^−2^; 2014, 21.8 °C and 296.4 W m^−2^) and afternoon (the combination of increased temperatures and high irradiation: 2013, 34 °C and 837.9 W m^−2^; 2014, 29.4 °C and 888.9 W m^−2^) at a flowering stage in rainfed or irrigated conditions. The measurement time (morning and afternoon conditions) had a statistically significant effect on all the tested parameters. The performance index (PI_ABS_) in 2013 and the maximum quantum yield of photosystem II (TR_0_/ABS) in 2014 are the only parameters significantly affected by the irrigation. As a result of the combined effect of increased temperatures and high irradiation, PI_ABS_ values decreased by 73–92% in rainfed conditions and by 63–87% in irrigated conditions in 2013, depending on the hybrid, while in 2014, the decrease varied between 70 and 86%. The TR_0_/ABS decrease was 7–17% in 2013, depending on the hybrid, and 6–12% in 2014, both in rainfed and irrigated conditions. The principal component analysis confirmed the effect of the combination of increased temperatures and high irradiation on hybrids, sorting them exclusively according to the time of measurement. All investigated parameters highly fluctuated between hybrids but without observable trends for the morning and afternoon conditions, as well as for irrigation. Plants’ reaction to the combination of increased temperatures and high irradiation manifested as a change in their photosynthetic efficiency, i.e., the photosynthetic apparatus’ functioning was impaired.

## 1. Introduction

Nowadays, scientists are increasingly researching the phenomenon of global warming and the associated climate change that is imposing abiotic stress on plants. Negative abiotic factors adversely affect plant growth and development, causing variation in the grain yield and crop quality of most economical crops, therefore, often limiting agricultural production worldwide [1,2]. Sunflower is one of the crops directly affected by weather condition changes. According to the botanical classification, sunflower is an annual plant that belongs to the kingdom *Plantae*, phylum *Magnoliophyta*, class *Magnoliopsida*, order *Asterales*, family *Asteraceae* (*Compositae*), genus *Helianthus*, and species *Helianthus annuus* L. [3]. The environment heavily influences its development, growth, and adaptation mechanisms. Changes in optimal temperatures, sunlight, and water availability during sunflower vegetation shorten certain developmental stages, impacting the final product, yield, and grain oil. Therefore, sunflower breeders have been focusing on creating genotypes that are adapted to non-optimal abiotic factors. Scientists predict that crop losses will occur in many regions of the world due to the more the frequent appearance of adverse weather conditions [4]. Some of the adverse weather conditions, such as lack of water [1], poorly distributed rainfall [5], and high temperatures [2], in conjunction with high irrigation in critical stages of plant development, are becoming more frequent nowadays. Global climate change leads to increased daily, seasonal, and annual mean air temperatures, uneven precipitation patterns, and changes in cloud coverage, in the intensity and quality of sunlight, and in the intensity and wind speed frequency [6]. One of the technologies used to mitigate the effects of environmental factors is irrigation. Implementing adequate irrigation is necessary for the context of climate change in any agricultural development strategy [7].

The stress effect on a plant depends on its survival potential, i.e., the assimilation and biomass accumulation, the plant’s developmental stage, and the stress factors’ duration [8]. The main reason for the occurrence of physiological and biochemical mechanisms that reduce plant productivity is oxidative stress caused by the excessive formation of reactive oxygen species (ROS). ROS disturb the balance between light energy absorption and its efficient utilisation of the photosynthetic process [9], invoking the need for antioxidant protection systems that reduce the possibility of oxidative damage. Such cell antioxidant protection systems include non-enzymatic antioxidants and stress enzymes whose primary role is to remove hydrogen peroxide [10]. 

Since environmental conditions highly influence photosynthesis, it is essential for agricultural production to understand the physiological processes affected by temperature, light, and water. For this reason, determining photosynthetic efficiency by measuring chlorophyll *a* fluorescence (ChlF) has been the topic of numerous studies. ChlF has often been used for determining the occurrence of stress through plants’ photosynthetic efficiency. ChlF is a small amount of energy in the form of light which is emitted during the return of the electron from excitation to the ground state. It provides information on the functioning of the electron transport chain in photosystem II [11]. Photosystem II is one of the most temperature and light-sensitive regulatory components through which the damage level of the photosynthetic apparatus is determined using the JIP test parameters. Understanding the mechanisms involved in plant responses to adverse environmental conditions is, without a doubt, the first step in creating crops with high-stress tolerance, which has proven to be a complex task in plant breeding [12]. 

Therefore, this study’s main objective was to compare changes in the photosynthetic efficiency that were induced by the combined effect of increased temperatures and high irradiation using chlorophyll fluorescence data recorded for different sunflower hybrids. We hypothesised that increased temperatures and high irradiation would reduce the photosynthetic efficiency in all the tested hybrids, as indicated by a decrease in ChlF. We also measured changes in the total and specific activity of the enzyme guaiacol peroxidase to explore their role in regulating the combined effect of increased temperatures and high irradiation responses of the selected hybrids. The same analyses were used for determining the impact of irrigation. Furthermore, principal component analysis (PCA) of the collected data enabled us to determine correlations for the tested traits and hybrids, showing their response to the combined effect of increased temperatures and high irradiation.

## 2. Results

### 2.1. Climate in the Experimental Site

**Figure 1:**
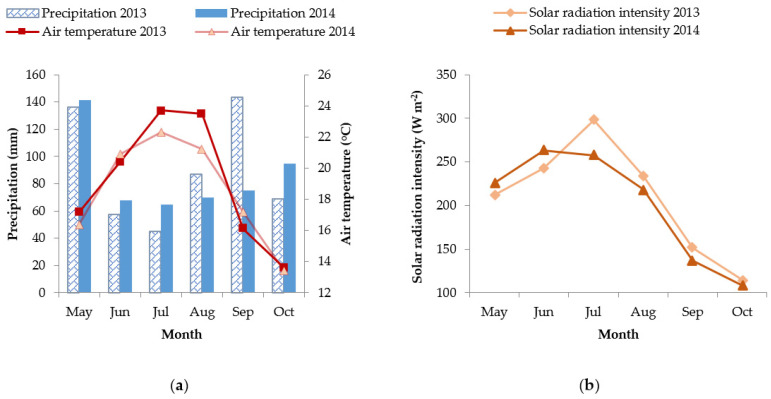
(**a**) Amount of precipitation (mm), mean air temperatures (°C), and (**b**) solar radiation intensity (W m^−2^) in 2013 and 2014.

The total amount of precipitation, the mean air temperatures, and the solar radiation intensity for the sunflower growing season in 2013 and 2014 were 537.7 mm and 514.1 mm; 19.1 °C and 18.6 °C; and 208.8 W m^−2^ and 201.7 W m^−2^, respectively. In 2013, the mean air temperatures were higher during the experiment, unlike in 2014 during which there was more precipitation (Figure 1). 

As expected, increases in afternoon air temperatures and solar radiation intensities were noted during the experiment in both years. The date of the measurements and sampling depended on the flowering and presence of clouds and rain. A weather station placed 270 m from the experiment recorded temperatures and intensities of solar radiation during the experiment. In 2013, the mean air temperature and solar radiation intensity during the morning conditions were 25.8 °C and 349.1 W m^−2^, respectively, and during afternoon conditions, they were 34 °C and 837.9 W m^−2^, respectively. In 2014, the mean air temperatures and solar radiation intensity during the morning conditions were 21.8 °C and 296.4 W m^−2^, respectively, and during the afternoon conditions, they were 29.4 °C and 888.9 W m^−2^, respectively. 

### 2.2. Chlorophyll a Fluorescence

**Figure 2:**
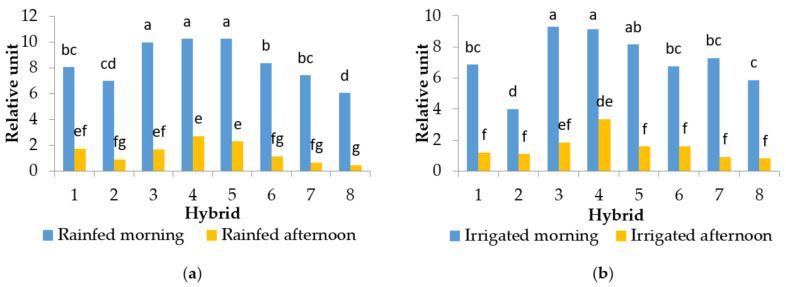
Mean values of the performance index (PI_ABS_) in 2013 per hybrid depending on the treatments and the time of measurement: (**a**) rainfed in the morning and afternoon conditions, and (**b**) irrigated in the morning and afternoon conditions. Means with the same letter are not significantly different according to Tukey’s HSD test at *p* < 0.01.

**Figure 3:**
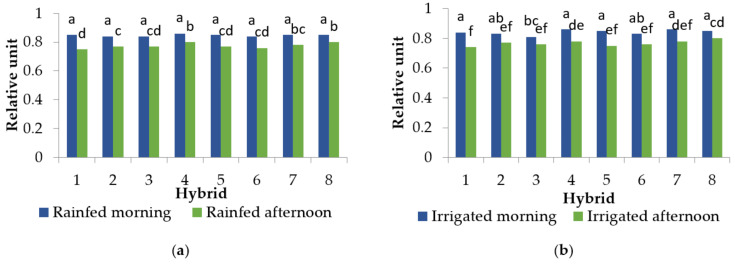
Mean values of the maximal quantum yield of photosystem II (TR_0_/ABS) in 2014 per hybrid depending on the treatments and the time of measurement: (**a**) rainfed in the morning and afternoon conditions, and (**b**) irrigated in the morning and afternoon conditions. Means with the same letter are not significantly different according to Tukey’s HSD test at *p* < 0.01.

The time of measurement (morning and afternoon conditions) had a statistically significant effect on all the tested parameters. Additionally, irrigation significantly affected the photosynthetic parameter performance index (PI_ABS_) in 2013 and the maximum quantum yield of photosystem II (TR_0_/ABS) in 2014. The hybrids had statistically different PI_ABS_ values within the measurement time and decreased PI_ABS_ due to the combined effect of the increased temperatures and high irradiation in 2013 (Figure 2). The decrease was approximately 73–92% in rainfed conditions and 63–87% in irrigated conditions, depending on the hybrid. There was no variability among hybrids in their TR_0_/ABS values in the morning conditions (Table 1). In 2014, only hybrid 3 had statistically different TR_0_/ABS values compared to the other hybrids (Figure 3). Values of TR_0_/ABS decreased in all the tested hybrids when plants were subjected to the combination of increased temperatures and high irradiation in both years, but irrigation affected TR_0_/ABS only in 2014 (Table 1 and Figure 3). The decrease in the value of TR_0_/ABS in 2013 was approximately 7–17%. In 2014, in the rainfed and irrigated conditions, the value of TR_0_/ABS decreased approximately by 6–12%.

Significant differences were found between hybrids in most JIP-test parameters in the morning and afternoon conditions (Table 1). 

In 2013, the combination of increased temperatures and high irradiation also influenced the absorption per active reaction centre (ABS/RC), increasing the values in hybrids 1 (18%), 2 (25%), 3 (23%), 5 (22%), 6 (20%), 7(33%), and 8 (30%), while in hybrid 4, values remained the same compared to the morning values. Trapping per active reaction centre (TR_0_/RC) had 5–11% higher values during the increased temperatures and high irradiation. Still, statistical differences were confirmed only for hybrids 5, 7, and 8, unlike other hybrids whose values were approximately similar to those measured in the morning conditions. Furthermore, the combination of increased temperatures and high irradiation caused a decrease of 13–38% in the electron transport per active reaction centre (ET_0_/RC), while dissipation per active reaction centre (DI_0_/RC) was increased (59–150%) in all the tested hybrids (Table 1).

In 2014 the combination of increased temperatures and high irradiation significantly affected ABS/RC, increasing the values between 7 and 24% in all the tested hybrids. A statistically significant difference was confirmed for TR_0_/RC in hybrids 1, 4, 5, and 8, with elevated values in the afternoon conditions of approximately 8, 11, 10, and 17%, respectively. In contrast, ET_0_/RC values decreased by 5–34% in the combined conditions of increased temperatures and high irradiation compared to the morning values. Furthermore, DI_0_/RC had an increase of approximately 48–104% depending on the hybrid, while PI_ABS_ had lower values of approximately 70–86% in all the tested hybrids due to the combination of increased temperatures and high irradiation (Table 1). 

### 2.3. Relative Water Content

When looking at the results of the relative water content (RWC) in 2013, it can be noted that RWC was significantly influenced by the combination of increased temperatures and high irradiation, compared to the morning conditions, only in hybrid 6 (Table 2). The value of RWC in hybrid 6 decreased by approximately 11%.

A reduction in RWC values in 2014 caused by the combination of increased temperatures and high irradiation was significant only in hybrid 1 in 2014 and was approximately 14% lower (Table 2). 

### 2.4. Enzime Guaiacol Peroxidase

The combined effect of the increased temperatures and high irradiation significantly reduced the total activity of the enzyme guaiacol peroxidase (GPOX) and the specific activity of guaiacol peroxidase (GPOXs) in most of the tested hybrids in 2013. The reduction of GPOX and GPOXs, depending on the hybrid, was between 15 and 39%, and between 4 and 42%, respectively. GPOX was not significantly different in hybrid 4 and there was no significant change in the GPOXs activity in hybrids 2 and 3 (Table 2).

GPOX activity in 2014 was increased under the influence of stress in hybrids 1 (176%) and 5 (148%), while the GPOXs activity significantly decreased in hybrids 2 (40%) and 7 (59%), and increased in hybrid 5 (102%) (Table 2).

### 2.5. Principal Component Analysis

**Figure 4:**
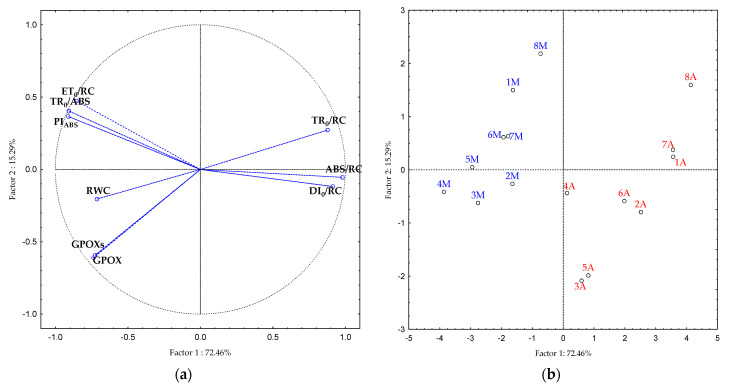
Biplots are constructed based on the results of the principal component analysis for thirteen parameters (**a**) and eight sunflower hybrids (**b**). M (blue) indicates measurements in the morning and A (red) indicates measurements in the afternoon conditions in the hybrid (1–8) view. Abbreviations: TR_0_/ABS—maximum quantum yield of photosystem II; ABS/RC—absorption per active reaction centre; TR_0_/RC—trapping per active reaction centre; ET_0_/RC—electron transport per active reaction centre; DI_0_/RC—dissipation per active reaction centre; PI_ABS_—performance index; RWC—relative water content; GPOX—total activity of the enzyme guaiacol peroxidase; and GPOXs—specific activity of guaiacol peroxidase.

Principal component analysis (PCA) was used to evaluate the combination of increased temperature and high irradiation effects in plants to identify the most sensitive parameters and hybrids. PCA provides information on the relationship between tested parameters and hybrids, i.e., similarity and variability. Of the main principal components, two (PC 1 and PC 2) explained 87.8% of the variability between the parameters and hybrids. The first principal component explained 72.5% of the total variability and the second explained 15.3%. The biplot (Figure 4a,b) shows how the correlations distributed the parameters and hybrids. The resulting positions in biplot squares show the influence of a particular parameter on the hybrid positions. The first biplot square (both PCs are positive) included TR_0_/RC and hybrids 1, 7, and 8 in the afternoon conditions. The second square (negative part of PC 1 axis and positive part of PC 2 axis) included ET_0_/RC, TR_0_/ABS, and PI_ABS_ with hybrids 1, 5, 6, 7, and 8 in the morning conditions. The third square (negative part of PC 1 and PC 2 axis) included the total GPOX activity, the specific GPOX activity, RWC, and hybrids 2, 3, and 4 in the morning conditions. Finally, the fourth square included ABS/RC, DI_0_/RC, and hybrids 2, 3, 5, and 6 in the afternoon conditions. 

## 3. Discussion

The intensity of the solar radiation, the air temperature, and the amount of available water are some environmental factors that are required for the plant to grow, develop, and perform physiological processes [13,14,15]. In this investigation, chlorophyll *a* fluorescence (ChlF) transients were measured to analyse the changes in the light phase of photosynthesis during the combined effect of increased temperatures and high irradiation in rainfed or irrigated conditions in sunflower plants. In many similar studies, the method of the ChlF measurement has proven to be the right choice for the determination of stress effects [16,17,18], which was also confirmed in this research study. 

Under ideal conditions, the optimal values of TR_0_/ABS for C3 plants, to which sunflower belongs to, are 0.83–0.84 and for C4 plants the value is 0.78 [19]. According to previous data, the photosynthetic apparatus is effective when TR_0_/ABS values range between 0.75 and 0.85 [20], thus it can be concluded that half of the tested sunflower hybrids in 2013 and hybrid 1 in 2014 had disturbed photosynthetic processes under the combination of increased temperatures and high irradiation (Figure 3 and Table 1). The remaining hybrids had reduced TR_0_/ABS in the afternoon conditions but those values did not go below 0.75, emphasising the impairment of the photochemical efficiency of photosystem II. The decrease in TR_0_/ABS values caused by the combination of increased temperatures and high irradiation has been reported previously [16,18,21,22]. Such TR_0_/ABS changes as a result of the combination of increased temperatures and high irradiation conditions may point to the deactivation or damage of the reaction centres of the photosystem II, i.e., photoinhibition [23]. In addition to TR_0_/ABS, parameter PI_ABS_ has been considered a better indicator of plant vitality and physiological status under stressful conditions due to its ability of early detection of changes in plants [24]. For this reason, the PI_ABS_ under stressful conditions has been investigated in numerous plant species such as maise (*Zea mays* L.) [25], wheat (*Triticum aestivum* L.) [26], Chinese cabbage (*Brassica rapa* L. *pekinesis*), white cabbage (*Brassica oleracea* var. *capitata*), kale (*Brassica oleracea* var. *acephala*) [17], fig (*Figus carica* L.) [16], cherry (*Prunus cerasus* L.) [27], and many others. Here, in the morning conditions, PI_ABS_ values were higher in the rainfed and irrigated treatments for all the tested hybrids in 2013 compared to the values in the combined conditions of increased temperatures and high irradiation (Figure 3). In 2013, the differences between rainfed and irrigated treatments were confirmed for PI_ABS_ but not for TR_0_/ABS; in 2014, it was the opposite. This could be explained by the higher mean air temperature in the afternoon conditions in 2013 (34.6 °C), which had a lower amount of available water, causing more significant changes in the plant vitality and photosynthetic status than in 2014, when the mean air temperature was 30 °C and plants had a better water supply. However, the solar radiation intensity was higher (888.9 W m^−2^) than in 2013 (837.8 W m^−2^) (Figure 1). Furthermore, these results confirmed that moisture conditions significantly influence the photosynthesis rate [28], but in this case, the influence was shown only for some ChlF parameters. 

The effect of the combination of increased temperatures and high irradiation on the energy transfer per active reaction centre of the photosystem II determined by the parameters ABS/RC, TR_0_/RC, ET_0_/RC, and DI_0_/RC is shown in Table 1. The ABS/RC indicates the ratio of chlorophyll molecules capable of absorbing energy and the number of active reaction centres (RCs) by which the functional size of the antenna complex is estimated [29]. In both tested years, the combination of increased temperatures and high irradiation significantly increased the values of ABS/RC, indicating the increased size of the apparent antenna or inactivation of a fraction of active RCs [24]. The increased ABS/RC, accompanied by an increased trapping per RC (TR_0_/RC) through the thylakoid membranes, is due to the high proton gradient, resulting in the transformation of RCs to “silent” RCs [25]. TR_0_/RC indicates the rate at which the reaction centre captures the exciton, causing the reduction of Q_A_ to Q_A^−^_ [11]. DI_0_/RC represents the ratio of the total amount of light dissipated to the number of active RCs that increase due to the high dissipation of inactive RCs [11]. The higher number of inactive RCs leads to more photons that the RCs cannot capture and excess energy is dissipated in the form of heat. In this study, the combination of increased temperatures and high irradiation increased DI_0_/RC due to the high dissipation of inactive RCs, which is associated with the decrease in ET_0_/RC, i.e., with the decrease in the ability to reduce the behind the primary electron acceptor (Q_A_). Energy dissipation is a defence mechanism that protects the plant against photo-oxidative damage by releasing heat energy. Furthermore, the results of this study suggest that, under the combination of increased temperatures and high irradiation, inactive RCs are associated with a decrease of TR_0_/ABS. The same pattern of the parameters per RC under heat and light stress was also confirmed by Mihaljević et al. [18] in two apple cultivars and by Mlinarić et al. [16] in figs.

Another physiological parameter used in this study was relative water content (RWC) in leaves. It is considered a quick and inexpensive laboratory method that breeders have included in their breeding programs as a technique by which they evaluate hybrids with the potential for tolerance or susceptibility to drought [30]. In this investigation, RWC in sunflower hybrids, for which the effect of the combination of increased temperatures and high irradiation was confirmed (hybrid 6 in 2013 and hybrid 1 in 2014), was lower than RWC in hybrids that showed better tolerance to these factors (Table 2). These results coincide with Popescu et al. [28] who confirmed maximum RWC values in grapevine (*Vitis vinifera* L.) in the morning and a reduction during the day. Such a different response of the hybrids to environmental conditions was also confirmed by Sairam et al. [31] in a study conducted during the sunflower maturation stage. 

The effect of the total activity of the enzyme guaiacol peroxidase (GPOX) has been examined, as well. GPOX belongs to the enzymatic antioxidant system of protection, whose purpose is to reduce the possibility of oxidative damage in plants. Its activity is highly dependent on the plant species and the causative of stress [32]. GPOX enzyme activity increased in all the hybrids in the combined conditions of increased temperatures and high irradiation in 2014 (Table 2). The same was reported in numerous other studies [16,33,34,35]. Results in 2013 had a different trend, as GPOX activity decreased in the afternoon conditions (Table 2). One possible reason for this decrement is the presence of high concentrations of H_2_O_2_. GPOX is one of the most important enzymes regulating intracellular H_2_O_2_ levels by removing H_2_O_2_ at lower concentrations [36]. Thus, it is possible that in this study, other enzymes of the antioxidant system were included in the degradation of ROSs. Another possible reason is the presence of high light intensity which, according to Lu et al. [37], can reduce the capacity of peroxidase for the removal of H_2_O_2_. However, the GPOX enzyme can remove ROSs more effectively than catalase because it has a higher affinity for H_2_O_2_ [10]. In contrast, increased GPOX activity suggests a better antioxidant response that successfully eliminates excess ROS [38]. In addition to the reduced total GPOX activities in 2013, the present study has also demonstrated a decline in the value of specific GPOX activities (GPOXs) in all sunflower hybrids during the combination of increased temperatures and high irradiation in 2013 and half of them in 2014 (Table 2). 

Other researchers have confirmed the influence of added water on the ChlF, RWC [27], and GPOX [39], which was not the case in this study (except for PI_ABS_ in 2013 and TR_0_/ABS in 2014). There was no statistically significant impact of irrigation, most probably because the amount of water was insufficient to make a difference. Nevertheless, the goal of this research study was not to determine sufficient irrigation water but to investigate the effect of mandatory irrigation before sunflower flowering. Conversely, the obtained results can be ascribed to the morphological structure of the sunflower root, which has the possibility of in-depth development, giving it better access to water from deeper layers of the soil [15].

PCA analysis clearly distinguished the position of the hybrids in the biplot (Figure 4). Hybrids were grouped exclusively according to the time of measurement, i.e., the morning conditions and conditions with the combination of increased temperatures and high irradiation. Pavlović et al. [17] showed similar results of a PCA analysis that separated crop species in control from cultivars in stressful conditions and had a similar grouping of parameters. The hybrids placed along the right side of the biplot were mostly determined by trapping, absorption, and dissipation per RC. On the other side of the biplot, PI_ABS_, TR_0_/ABS, and ET_0_/RC were grouped with GPOX, GPOXs, and RWC. Similar grouping in the PCA analysis was confirmed in the studies of Pavlović et al. [17] and Viljevac Vuletić et al. [40].

## 4. Materials and Methods

### 4.1. Plant Materials and Plant Growing Conditions

Eight sunflower (*Helianthus annuus* L.) hybrids were chosen for study based on the differences in the pedigree and agronomic properties (plant height, head diameter, grain and oil yield, and oil content). Two hybrids were standards and six hybrids were developed at the Agricultural Institute Osijek. The study was conducted in 2013 and 2014 under field conditions (45°32′ N, 18°44′ E). The experiment was set up in two treatments and four replications. The first treatment was rainfed, while in the second treatment, the hybrids were irrigated as needed to prevent water deficit conditions. The distance between treatments was 5 m. All management practices in the experiment were carried out according to the recommendations and requirements of sunflower cultivation. Each hybrid was sown by a hand planter (two seeds per hill) at a 4 cm depth in four 5 m long rows, with a distance between the rows of 70 cm and a distance within rows of 23 cm, in a sandy clay loam soil. Measurements were made at the flowering stage (R5.3 to R5.8 stage determined according to Schneiter and Miller [41]). The plants were covered with polyethene meshes after flowering to protect the experiment from birds.

### 4.2. Irrigation of the Experimental Field

The irrigation of the experiment was carried out with a travelling sprinkler system. The irrigation system covered 28 to 30 m, with an efficiency of irrigation of about 95%. The water used for irrigation was pumped by an electric pump (5.5 kW) from a 37 m deep well and it satisfied the required quality parameters as recommended for sunflower cultivation. Granular matrix sensors (Watermark Model 200SS, Watermark Model 200SS, Riverside, CA, USA) were used for determining the soil moisture and consequently the initial irrigation time on which the irrigation scheduling was based. Before installation, the sensors were calibrated based on the soil water content by the gravimetric method. The soil retention capacity was at 37% vol. In both years, sensors were placed in both treatments at a 10–15 cm and 25–30 cm depth after emergence. The water content was recorded twice a week during the experiment with a digital soil moisture meter (Watermark, Irrometer Company, Inc. Riverside, CA, USA). During 2013, four irrigations were carried out (beginning of stem elongation, butonisation, before flowering, and full flowering), with 97 mm of water added during the season, while only one irrigation was carried out (before flowering) in 2014 with 25 mm of water added. One of the irrigations in both years was carried out just before the flowering of the hybrid, according to a recommendation by Lisogorov [42]. All irrigation applications occurred in the early morning or late evening to prevent plant damage. Irrigation was initiated when the granular matrix sensor values were about 80 cbars or when the soil water content fell below 60% of the field water capacity. Field water capacity was previously determined to be at 37% vol. The mean specific density of the soil was 1.40 g/cm^3^ and the wetting depth was 30 cm.

### 4.3. Field Measurements and Tissue Sampling

All analyses were conducted on the upper (youngest) developed leaves per hybrid during flowering. According to Schneiter and Miller [41], a leaf is considered developed if larger than 4 cm. The ChlF measurements and sampling of the plant material were done during the mornings (7:30–9:00 a.m.) and early afternoons (12:30–2:00 p.m.) in the middle two rows of each sunflower hybrid in the field. The measurements and tissue samplings in the morning hours represented control conditions, while the measurements and tissue samplings in the afternoon hours represented a combination of increased temperatures and high irradiation. ChlF was determined in the field on three randomly selected plants per hybrid in each repetition (12 measurements per hybrid). The two upper (youngest) developed sunflower leaves per hybrid (2 leaves × 4 replicates), on which photosynthetic efficiency was determined, were sampled for laboratory analyses. The composite sample made from eight leaves per hybrid was used for all analyses, except for determining the RWC, for which five randomly selected leaves per hybrid were used. Before the biochemical analysis, plant materials were homogenised into a powder using liquid nitrogen. All laboratory analyses were done in five biological replications.

### 4.4. Chlorophyll a Fluorescence (ChlF)

ChlF was determined using the Plant Efficiency Analyser (Handy PEA, Hansatech, UK). Sunflower leaves were adapted to dark with special leaf clips for a minimum of 30 min before measuring, after which ChlF transients were induced using a pulse of saturating red light (peak at 650 nm, 3200 μmol m^−2^ s^−1^). OJIP transients were measured by recording data from 50 μs to 1 s. Recorded fluorescence data were used for calculating JIP parameters, according to Strasser et al. [11]. In this study, the following parameters were determined: TR_0_/ABS (maximum quantum yield of photosystem II), ABS/RC (absorption per active reaction centre), TR_0_/RC (trapping per active reaction centre), ET_0_/RC (electron transport per active reaction centre), DI_0_/RC (dissipation per active reaction centre), and PI_ABS_ (performance index), expressed in relative units.

### 4.5. Relative Water Content (RWC)

The fresh weight (FW) was determined from approximately 1 cm^2^ of fresh sunflower leaf. The tissue was stored in distilled water in a refrigerator at 8 °C for four hours. After saturation, the tissue was dried with cellulose, weighed to determine its turgid weight (TW), and then was placed in an oven at 80 °C for 24 h to determine the dry weight (DW). The RWC was calculated according to the formula by Poormohammad Kiani et al. [43]: RWC (%) = (FW − DW)/(TW − DW) × 100.

### 4.6. Enzime Guaiacol Peroxidase 

The total activity of the enzyme guaiacol peroxidase (GPOX; EC 1.11.1.7) was measured according to Siegel and Galston [44] from crude protein extract. Before analysis, a composite sample of sunflower leaves was powdered in liquid nitrogen with the addition of polyvinylpolypyrrolidone (PVP) using a pestle and mortar. Crude proteins were extracted from 0.2 g of powdered tissue with 1 mL of 100 mM potassium phosphate buffer, pH 7.0, for 15 min on ice. After centrifugation for 15 min at 14,000× *g* and 4 °C, re-extraction with 1 mL of the same buffer was performed. The joint supernatant was used for spectrophotometrical determination of GPOX activity. The reaction mixture (pH 5.8) contained 5 mM of guaiacol, 0.2 M of KH_2_PO_4_, 0.2 M of Na_2_HPO_4_ × 12 H_2_O, and 5 mM of H_2_O_2_. The enzymatic reaction was started by adding 40 μL of crude protein extract to the reaction mixture. The increase in absorbance was monitored at 470 nm every second for one minute and was expressed as min^−1^ g^−1^ of fresh mass. The protein concentration in the crude protein extract was determined according to Bradford [45] and expressed in mg g^−1^. The specific activity of guaiacol peroxidase (GPOXs) was determined as a quotient of the total GPOX activity and protein concentration. GPOXs were expressed as a change in the absorbance at 470 nm min^−1^ mg^−1^ protein. 

### 4.7. Data Analyses

The general linear model (GLM) was used for determining differences between the measured data at the *p* < 0.01 level using Statistica 8.0 software (StatSoft, Inc. 2007). The sources of variability were hybrid, treatment (rainfed and irrigated), replications, year (2013 and 2014), and time of measurement (morning and afternoon conditions). Since there were no significant differences between replications and treatments, all ChlF parameters (except for PI_ABS_ in 2013 and TR_0_/ABS in 2014) as well as physiological and biochemical data were pooled to produce statistic tests (Figure 2 and Figure 3, and Table 1 and Table 2) in order to find significant differences between hybrids in the morning and afternoon conditions. Tukey’s post-hoc honest significant difference (HSD) test was used to compare the mean values of the tested parameters at the *p* < 0.01 level. The analysis of variance was used for establishing differences between hybrids per time of measurement (morning and afternoon conditions) for PI_ABS_ in 2013 and TR_0_/ABS in 2014 with *p* < 0.01. The data presented in the text, tables, and figures are means of 24 replications (*n* = 24) for fluorescence measurements (except for PI_ABS_ in 2013 and TR_0_/ABS in 2014 when there were 12 replications per treatment) and means of 20 replications (*n* = 10) for RWC, GPOX, and GPOXs. Principal component analysis (PCA) was used for correlations among the measured physiological and biochemical parameters. Values for treatments, the time of measurement, and years were pooled and used for PCA.

## 5. Conclusions

This study confirmed the variable sensitivity of hybrids to environmental factors caused by increased temperatures, high irradiation, and differences in the reaction to irrigation by analysing biochemical parameters. All the investigated parameters had large value fluctuations and lacked a clearly observable general regularity in the morning and afternoon conditions and irrigation. Plants reacted to the combination of increased temperatures and high irradiation with changes in their photosynthetic efficiency, which impaired the functioning of the photosynthetic apparatus. No evident trend was found for the relative water content, the leading indicator of the combination of increased temperatures and high irradiation. Values for the total and specific activity of the enzyme guaiacol peroxidase suggest that guaiacol peroxidase is not the primary antioxidant enzyme in sunflower leaves responsible for cell defence under the investigated conditions. 

## Figures and Tables

**Table 1:** Mean values of JIP-test parameters (*n* = 24) for eight sunflower hybrids in the morning and afternoon conditions in 2013 and 2014. Means with the same letters in columns are not significantly different according to Tukey’s HSD_0.01_.

Hybrid	Parameter/ Year/ Condition	TR_0_/ABS	ABS/RC		TR_0_/RC		ET_0_/RC		DI_0_/RC		PI_ABS_
		2013	2013	2014	2013	2014	2013	2014	2013	2014	2013
1	M	0.84 ^a^	1.83 ^ghi^	1.81 ^fg^	1.54 ^efgh^	1.54 ^bcd^	1.11 ^b^	1.11 ^ab^	0.29 ^ef^	0.28 ^fgh^	8.03 ^d^
	A	0.75 ^cd^	2.16 ^cde^	2.24 ^a^	1.61 ^cde^	1.66 ^a^	0.79 ^e^	0.73 ^g^	0.56 ^cd^	0.57 ^a^	1.12 ^g^
2	M	0.82 ^a^	1.94 ^efghi^	1.85 ^fg^	1.59 ^defg^	1.54 ^bcd^	1.07 ^bc^	1.12 ^ab^	0.35 ^ef^	0.31 ^fg^	7.55 ^d^
	A	0.71 ^ef^	2.42 ^bc^	1.98 ^cde^	1.70 ^cd^	1.52 ^cd^	0.80 ^e^	0.77 ^fg^	0.72 ^ab^	0.46 ^cd^	1.85 ^fg^
3	M	0.85 ^a^	1.69 ^i^	1.82 ^fg^	1.43 ^h^	1.51 ^cd^	1.06 ^bc^	1.11 ^ab^	0.26 ^f^	0.31 ^f^	7.69 ^d^
	A	0.75 ^c^	2.07 ^defg^	2.06 ^bc^	1.56 ^defgh^	1.57 ^abc^	0.83 ^de^	0.83 ^fg^	0.51 ^cd^	0.49 ^bc^	1.92 ^fg^
4	M	0.85 ^a^	1.73 ^hi^	1.59 ^hi^	1.46 ^fgh^	1.36 ^e^	1.10 ^b^	1.03 ^bc^	0.27 ^f^	0.23 ^h^	11.55 ^a^
	A	0.79 ^b^	1.99 ^defgh^	1.92 ^def^	1.56 ^defgh^	1.51 ^cd^	0.96 ^cd^	0.85 ^ef^	0.43 ^de^	0.41 ^de^	2.68 ^ef^
5	M	0.84 ^a^	1.73 ^hi^	1.75 ^g^	1.45 ^gh^	1.48 ^cd^	1.09 ^bc^	1.11 ^fgh^	0.28 ^f^	0.27 ^fgh^	9.46 ^b^
	A	0.76 ^bc^	2.11 ^def^	2.14 ^ab^	1.59 ^def^	1.63 ^ab^	0.88 ^de^	0.77 ^fg^	0.51 ^cd^	0.52 ^ab^	1.39 ^g^
6	M	0.83 ^a^	1.83 ^ghi^	1.83 ^fg^	1.52 ^efhg^	1.53 ^bcd^	1.10 ^b^	1.13 ^a^	0.31 ^ef^	0.30 ^fg^	8.14 ^cd^
	A	0.74 ^cde^	2.20 ^cde^	2.04 ^bcd^	1.61 ^cde^	1.56 ^bcd^	0.82 ^e^	0.80 ^fg^	0.59 ^bc^	0.49 ^bc^	1.72 ^fg^
7	M	0.84 ^a^	1.87 ^fghi^	1.72 ^gh^	1.57 ^defg^	1.47 ^cd^	1.13 ^b^	1.07 ^abc^	0.30 ^ef^	0.25 ^gh^	9.12 ^bc^
	A	0.70 ^f^	2.49 ^b^	2.02 ^bc^	1.74 ^c^	1.57 ^abc^	0.77 ^e^	0.79 ^fg^	0.75 ^a^	0.45 ^cd^	1.90 ^fg^
8	M	0.85 ^a^	2.25 ^bcd^	1.54 ^i^	1.91 ^b^	1.31 ^e^	1.35 ^a^	0.98 ^cd^	0.34 ^ef^	0.23 ^h^	11.33 ^a^
	A	0.72 ^def^	2.92 ^a^	1.90 ^ef^	2.09 ^a^	1.53 ^bcd^	0.84 ^de^	0.93 ^de^	0.84 ^a^	0.38 ^e^	3.39 ^e^

Abbreviations: TR_0_/ABS—maximum quantum yield of photosystem II; ABS/RC—absorption per active reaction centre; TR_0_/RC—trapping per active reaction centre; ET_0_/RC—electron transport per active reaction centre; DI_0_/RC—dissipation per active reaction centre; PI_ABS_—performance index; M—morning; and A—afternoon.

**Table 2:** Mean values of the physiological and biochemical parameters (*n* = 20) for eight sunflower hybrids in the morning and afternoon conditions in 2013 and 2014. Means with the same letters in columns are not significantly different according to Tukey’s HSD_0.01_.

Hybrid	Parameter/ Year/ Condition	RWC		GPOX		GPOXs	
		2013	2014	2013	2014	2013	2014
1	M	65.86 ^bcd^	73.73 ^a^	57.51 ^ef^	5.45 ^i^	1.49 ^bcd^	0.25 ^g^
	A	66.25 ^bcd^	63.81 ^c^	36.82 ^hi^	15.03 ^fg^	0.86 ^ghi^	0.43 ^efg^
2	M	73.18 ^a^	69.10 ^abc^	63.21 ^cde^	19.68 ^cde^	1.03 ^fghi^	1.59 ^a^
	A	68.50 ^abc^	68.51 ^abc^	43.81 ^gh^	22.23 ^bcde^	0.74 ^hi^	0.95 ^bcd^
3	M	66.79 ^bcd^	67.68 ^abc^	87.38 ^a^	20.25 ^bcde^	1.76 ^abc^	0.91 ^bcde^
	A	66.83 ^bcd^	68.85 ^abc^	73.84 ^bc^	24.22 ^ab^	1.69 ^abc^	0.86 ^bcdef^
4	M	66.83 ^bcd^	72.56 ^ab^	71.33 ^cd^	22.59 ^abcd^	1.92 ^a^	0.94 ^bcd^
	A	64.45 ^cde^	71.46 ^ab^	60.84 ^def^	23.26 ^abc^	1.23 ^def^	0.50 ^defg^
5	M	68.28 ^abc^	71.22 ^ab^	84.12 ^ab^	10.78 ^gh^	1.78 ^ab^	0.52 ^cdefg^
	A	70.95 ^ab^	71.22 ^ab^	61.86 ^de^	26.78 ^a^	1.43 ^cde^	1.05 ^b^
6	M	72.93 ^a^	70.49 ^ab^	65.59 ^cde^	17.76 ^ef^	1.12 ^efg^	0.64 ^bcdefg^
	A	65.10 ^cde^	70.06 ^abc^	41.49 ^ghi^	21.31 ^bcde^	0.71 ^i^	1.12 ^ab^
7	M	64.27 ^cde^	72.86 ^ab^	64.43 ^cde^	18.48 ^def^	1.07 ^fgh^	1.02 ^bc^
	A	59.75 ^e^	68.67 ^abc^	39.38 ^ghi^	19.11 ^cdef^	0.72 ^i^	0.42 ^efg^
8	M	66.86 ^bcd^	70.41 ^ab^	50.26 ^fg^	8.43 ^hi^	1.17 ^defg^	0.40 ^fg^
	A	61.54 ^de^	66.98 ^bc^	32.72 ^i^	9.67 ^hi^	0.74 ^hi^	0.47 ^defg^

Abbreviations: RWC—relative water content (%); GPOX—total activity of the enzyme guaiacol peroxidase (ΔA_470_ min^−1^ g^−1^); GPOXs—specific activity of guaiacol peroxidase (ΔA_470_ min^−1^ g^−1^ protein); M—morning; and A—afternoon.

## Data Availability

Not applicable.
